# Magnetic Field-Assisted Photocatalytic Degradation of Organic Pollutants over Bi_1−x_R_x_FeO_3_ (R = Ce, Tb; x = 0.00, 0.05, 0.10 and 0.15) Nanostructures

**DOI:** 10.3390/ma14154079

**Published:** 2021-07-22

**Authors:** Radhalayam Dhanalakshmi, Nambi Venkatesan Giridharan, Juliano C. Denardin

**Affiliations:** 1 Physics Department and CEDENNA, University of Santiago of Chile (USACH), Santiago 9170124, Chile; 2 Advanced Functional Materials Laboratory, Department of Physics, National Institute of Technology, Tiruchirappalli 620015, TN, India; giri@nitt.edu

**Keywords:** BiFeO_3_, hydrothermal, nanostructures, photocatalysis, phenol red, magnetic field

## Abstract

Magnetic-field-accelerated photocatalytic degradation of the phenol red (PR) as a model organic pollutant was studied using rare-earth elements modified BiFeO_3_ (Bi_1__−x_R_x_FeO_3_ (R = Ce, Tb; x = 0.0, 0.05, 0.10 and 0.15); BFO: RE) nanostructures. The nanostructures were prepared via the hydrothermal process and their morphological, structural, functional, optical and magnetic features were investigated in detail. The effect of magnetic fields (MFs) on photocatalysis were examined by applying the different MFs under visible light irradiation. The enhanced photodegradation efficiencies were achieved by increasing the MF up to 0.5T and reduced at 0.7T for the compositions x = 0.10 in both Ce and Tb substituted BFO. Further, mineralization efficiencies of PR, reproducibility of MF-assisted photocatalysis, stability and recyclability of BFO: RE nanostructures were also tested.

## 1. Introduction

Over the last decades, Bismuth ferrite (BFO) is one of the most efficient and promising materials that have gained much attention in modern scientific research because of its great prospect in resolving energy and environmental issues [1,2]. It was established as an attractive visible light photocatalyst due to its narrow bandgap (2.1 eV), advanced magnetic properties, excellent chemical stability, low cost and non-toxic [3,4,5]. BFO has been considered as an alternative for efficiency limits compared to widely used photocatalytic materials TiO_2_ and ZnO since these can only absorb ultraviolet light, which occupies only 4% of the whole solar energy [6,7]. The strong oxidation and reduction capabilities (1.74 V (vs. NHE) and −0.39 V (vs. NHE), respectively, [8]) of BFO can be used to convert light energy into different energy types and make changes in organic azo dyes such as phenol red. Phenol red (C_19_H_14_O_5_S) [9,10] is one of the groups of organic phenol dye (standard reduction potential value of 0.35V (vs. SCE) [11,12]), belongs to the family of triphenylmethane dyes. These are extremely brilliant and intensely colored synthetic organic dyes, used in the paper, textile industries, in the coloring of a large variety of products, as well as in the analytical and medical sectors because they can provide the whole coloration range [13]. These compounds are present in a high number of colored industrial wastewaters that are released into the environment. PR is one of harmful dye, which is widely used in chemical laboratories as a pH indicator, estrogenic properties and screening test. Despite its applications, toxicology information reveals that contact or prolonged exposure to PR may produce skin and eyes damage, irritation with redness and pain. The PR inhalation can cause irritation to the respiratory tract, and ingestion can affect the gastrointestinal tract. Hence, the choice of phenol red dye removal by BFO is of enormous value. However, BFO in its pure form shows low photocatalytic efficiency due to its rapid recombination of charge carriers. In this aspect, many strategies have been developed for improving photocatalytic efficiencies, such as doping with rare-earth (RE) elements [14], making heterojunctions, metal loading etc. Despite of these approaches, retarding the high recombination rate of photo-generated carriers is one of the effective approaches to enhance the catalytic performance. This could be realized by coupling photocatalysis with various external energy fields. Recently, photocatalytic efficiency has been significantly enhanced by using the external electric field [15,16,17] to the photocatalysis. For instance, an oriented electric field accelerates the non-redox, bond-forming process of Diels-Alder reaction [18] and evolves an opportunity of precise manipulation of chemical reaction. The catalytic activity of platinum can also tune by elastic strain in a regulated and predictable way [19,20]. In the similar way microwave [21,22], ultrasonic fields [23,24], low-temperature plasma [25,26] and magnetic fields (MFs) [27] have been used to acquire the beneficial effect on photocatalysis both for duration and efficiency. Among these, a MF-assisted photocatalytic process has been put forward to control the photo-induced hole and electron recombination, which is similar to the electron transfer reactions. Therefore, by considering the resemblances between photocatalytic and electron transfer reactions, the utilization of MFs to raise the photocatalytic efficiency, i.e., the effect of external MFs on chemical reactions is the MF-assisted photocatalytic activity [27,28], which is an environment-friendly method, and has been extensively discussed and efficiently contributed towards sustainable advancements [29].

BFO exhibits the coexistence of ferroelectric and magnetic order in the same phase. It has the rhombohedral distorted ABO_3_ type perovskite structure and possesses the G-type antiferromagnetism including cycloidal spin structure [30]. The enhanced magnetic properties of RE substituted BFO suppress its cycloidal spin structure, would affect the magnetic-field assisted photocatalytic activity of the BFO nanostructures [31]. An applied external MF may change the possible reaction probability and the movement of the charged species by magnetically induced retarding of electron-hole recombination [28]. However, the strength of the MF affects the recombination rate of the photo-generated radicals [32]. Strong MFs up to several kOe have been used for noticeable improvements in photocatalytic responses using TiO_2_ and ZnO catalysts. Wakasa et al. investigated the MF effects on photodegradation of tert-butyl alcohol with platinized TiO_2_ particles under 0–1.5T of an external MF [33]. Lately, an enhancement in the photocatalytic behavior of ZnO by applying the MFs were studied by Okumura et al. [34]. The enhanced photocatalytic activity observed through MF boosting carrier transport in α-Fe_2_O_3_/rGO [35]. However, to the best of our knowledge, the comparative studies on the MF-assisted photocatalytic activity of RE-substituted BFO (BFO: RE) under different field strengths towards the degradation of organic pollutant phenol red have not yet been made. In this work, we chosen the cerium and terbium as a model RE elements to investigate the MF-accelerated photocatalytic behavior of BFO. The hydrothermal process has been adapted to prepare the Bi_1−x_R_x_FeO_3_ (R = Ce, Tb; x = 0.00, 0.05, 0.10 and 0.15) nanostructures and studied their various properties. The enhanced MF-accelerated photocatalytic activity of Bi_1−x_R_x_FeO_3_ (R = Ce, Tb; x = 0.00, 0.05, 0.10 and 0.15) nanostructures were investigated by applying the different external MFs towards the degradation of PR in visible-light illumination. In order to evaluate the reproducibility of heterogeneous MF-assisted photocatalysis and stability of catalysts, the recyclable tests were also studied. 

## 2. Materials and Methods

The Bi_1−x_R_x_FeO_3_ (R = Ce, Tb; x = 0.00, 0.05, 0.10 and 0.15) nanostructures were synthesized at 200 °C by hydrothermal process [36,37] by using bismuth nitrate [Bi (NO_3_)_3_·5H_2_O], iron nitrate [Fe (NO_3_)_3_.9H_2_O], cerium nitrate [CeN_3_O_9_·6H_2_O] and terbium nitrate [Tb (NO_3_)_3_·6H_2_O] as precursors. The size and morphology of the prepared nanostructures were investigated using HR-TEM (Jeol 2010, Jeol, Tokyo, Japan). The purity and crystallinity of the samples were examined by X-ray diffractometry (XRD) on a (D/MAX Ultima III, Rigaku Corporation, Tokyo, Japan) with CuKα radiation. Fourier transform infrared (FT-IR) spectroscopy (Perkin Elmer/spectrum RXI, Perkin Elmer, Texas City, TX, USA) was used to evaluate the local structure of the samples. The optical studies of prepared nanostructures were conducted on a UV-visible diffuse reflectance spectrophotometer (JASCO Model V-670, Jasco, Easton, MD, USA). The magnetic properties of prepared samples were studied through vibrating sample magnetometry (VSM) (Lake Shore-7404 VSM, Lake Shore Cryotronics, Westerville, OH, USA).

The MF-assisted photocatalytic activity of Bi_1−x_R_x_FeO_3_ (R = Ce, Tb; x = 0.00, 0.05, 0.10 and 0.15) were studied by applying the external MFs of 0.0, 0.3, 0.5 and 0.7T. An electromagnet was used to produce the preferred MF to degrade the 10 ppm of PR (pH 6.7) solution, which is placed in a quartz cell along with 10 mg of the synthesized photocatalyst. Before illumination to the visible light, the solution was stirred for 30 min in dark to attain absorption and desorption equilibrium. The catalytic activities were carried out under the visible light irradiation by a 150 W Xenon lamp (Toption, Toption Instruments, Xian, China) with a cutoff filter for *λ* ≥ 400 nm (600 mW/cm^2^ at wavelength of 650 nm) at room temperature (30–32 °C). After the elapse of known period (for every 15mins), a small quantity of the solution was taken and centrifuged to remove any catalyst in the solution. The concentration of PR was determined by measuring the absorbance at 443 nm using a UV-Vis absorption spectrophotometer (Shimadzu UV-1700, Shimadzu, Kyoto, Japan). The mineralization efficiencies of PR were performed on a total organic carbon Analyzer (Analytikjena/multi-N/C 3100, Analytik Jena, Jena Germany), respectively.

## 3. Results and Discussion

**Figure 1:**
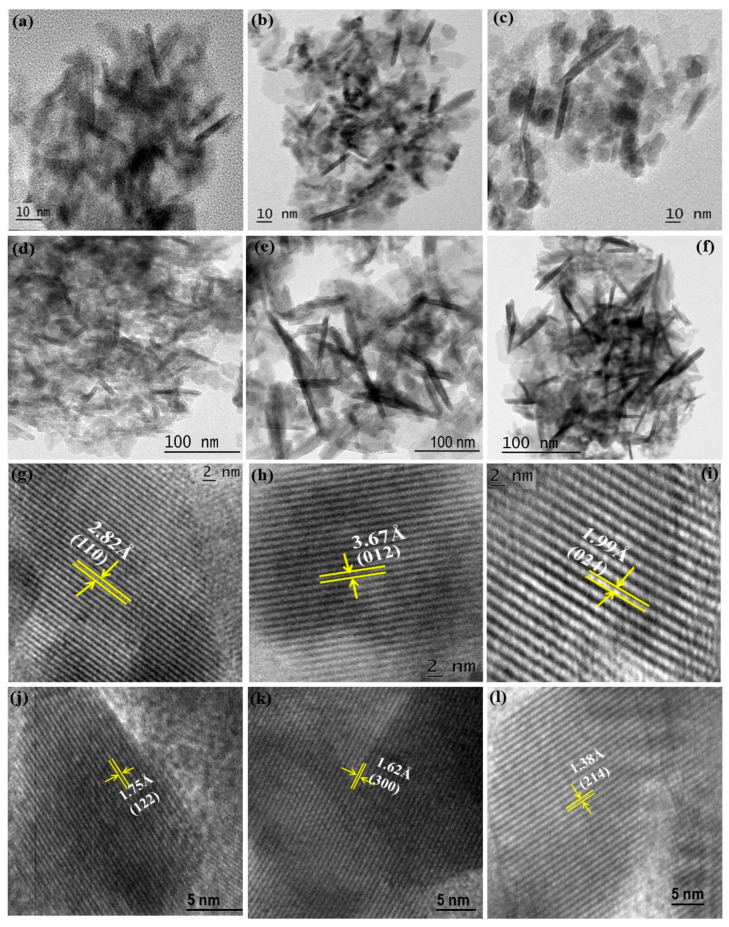
(**a**–**f**) HR-TEM images, (**g**–**l**) lattice fringes for the Bi_0.95_Ce_0.05_FeO_3_, Bi_0.90_Ce_0.10_FeO_3_, Bi_0.85_Ce_0.15_FeO_3_, Bi_0.95_Tb_0.05_FeO_3_, Bi_0.90_Tb_0.10_FeO_3_, Bi_0.85_Tb_0.15_FeO_3_ nanostructures.

Figure 1a–f shows the typical HR-TEM images of Bi_1−x_R_x_FeO_3_ (R = Ce, Tb; x = 0.05, 0.10 and 0.15) nanostructures. The nanostructures were found to have nano-rod-like morphology and seem similar to be agglomerated. The length and diameter of nano-rods were around 16–24 nm and 1.6–3.5 nm for BFO: Ce nanostructures, whereas the length and diameter of nanorods were observed as 20–30 nm and 4–6.5 nm for BFO: Tb nanostructures, respectively.

Figure 1g–l illustrates the lattice fringes corresponding to the Bi_1−x_R_x_FeO_3_ (R = Ce, Tb; x = 0.05, 0.10 and 0.15), revealed that phase formation occurred with a high order of crystalline nature. The distance between two parallel planes of Bi_1−x_Ce_x_FeO_3_ (x = 0.05, 0.10 and 0.15) nanostructures were found to be 2.82 Å (110), 3.67 Å (012) and 1.99 Å (024), respectively. For the BFO: Tb nanostructures, the lattice fringe spacing were observed as 1.75 Å (122), 1.62 Å (300) and 1.38 Å (214), respectively, in agreement with interplanar distance estimated from the XRD analysis. 

**Figure 2:**
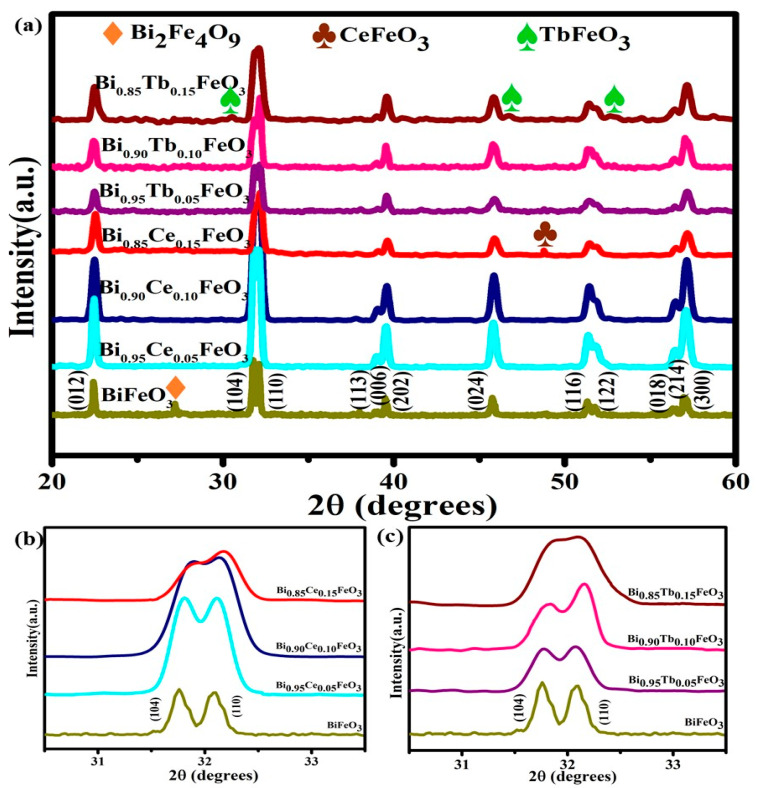
(**a**) XRD patterns of Bi_1−x_R_x_FeO_3_ (R = Ce, Tb; x = 0.00, 0.05, 0.10 and 0.15) samples synthesized at 200 °C. (**b**) Expanded XRD pattern for the BFO: Ce, (**c**) BFO: Tb samples.

Figure 2a shows the X-ray diffraction patterns of hydrothermally synthesized BFO: RE nanostructures. The diffraction peaks of x = 0.00 corresponds to the rhombohedral (*R3c* space group) of BFO. However, the secondary phase (Bi_2_Fe_4_O_9_) is also detected at 27° due to the kinetics of formation. This phase is well suppressed when the concentration of RE elements increased to 5% and 10%, which is consistent with the earlier reports on substitution with a moderate amount of RE ions in place of Bi^3+^ could eliminate the impurity phases in BFO [38,39,40,41]. However, by increasing the concentration of Ce and Tb to 15% (Bi_0.85_Ce_0.15_FeO_3_ and Bi_0.85_Tb_0.15_FeO_3_), the presence of the small impurity phases was assigned as the CeFeO_3_ and TbFeO_3_, respectively.

The magnified XRD patterns in the vicinity of 2θ = 32° are shown in Figure 2b,c. By increasing the concentration of RE, rhombohedral peak merging and shifting to the higher angles may reflect the structural transformation from rhombohedral (*R3c*) to orthorhombic ((*Pnma*) and (*Pn2*_1_*a*)) for BFO: Ce and BFO: Tb nanostructures, respectively, which are reliable with the earlier reports [39,40,41].

**Figure 3:**
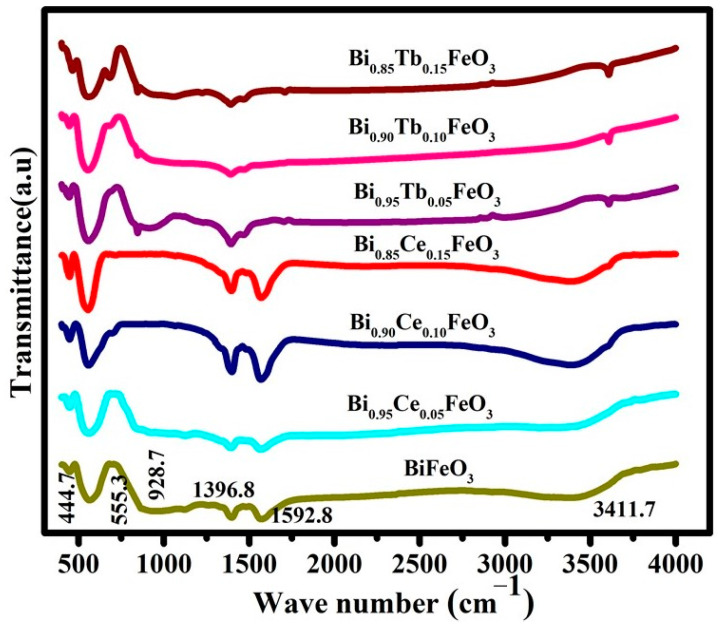
FT-IR spectrum of the as-prepared BFO: RE nanostructures.

The FTIR spectrum BFO: RE nanostructures are illustrated in Figure 3. The broad band between 3000–3600 cm^−1^ arose from the antisymmetric and symmetric stretching of bond H_2_O and OH^−^ groups, while a band at 1592 cm^−1^ corresponded to the bending vibrations of H_2_O [42,43]. Bands between 900 and 1410 cm^−1^ are due to the existence of trapped nitrates present in the sample. The presence of a metal-oxygen band can confirm the formation of the perovskite structure [44]. The formation of FeO_6_ octahedra of the perovskite structure by the typical characteristic metal-oxygen bonds between 400 and 600 cm^−1^ in all samples.

**Figure 4:**
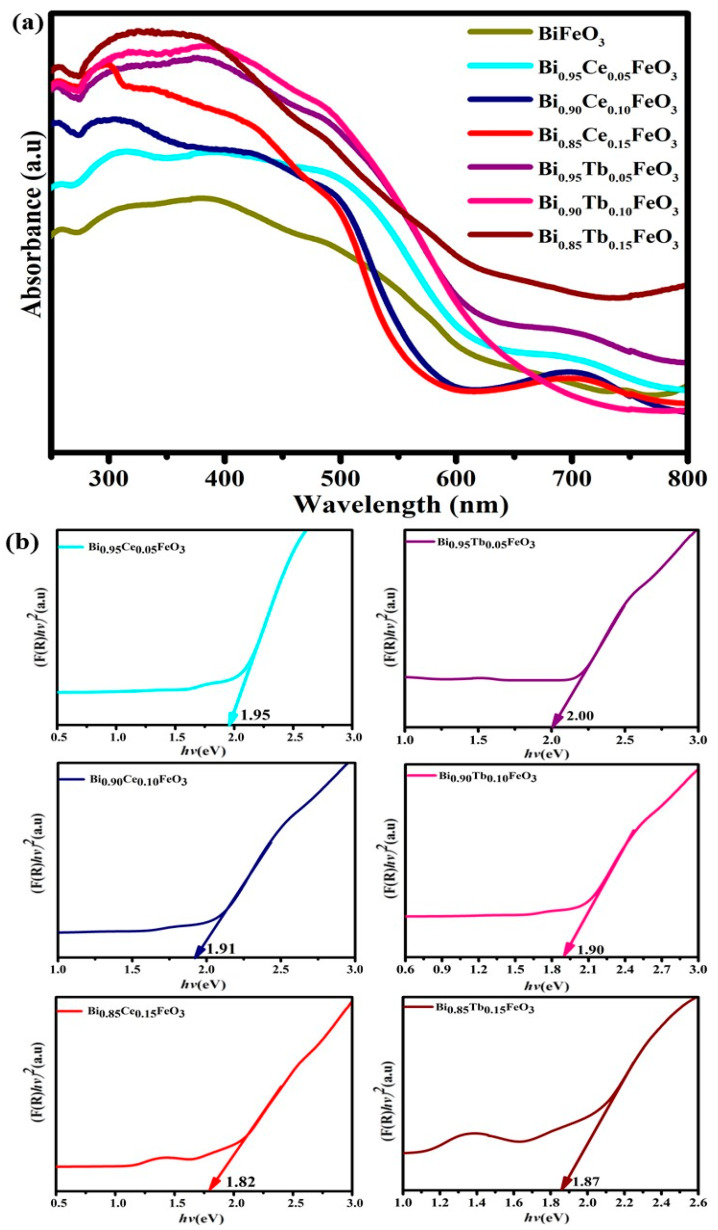
(**a**) DRS spectra of BFO: RE nanostructures. (**b**) (*F*(R)*hv*)^2^ versus photon energy (*hv*) for the Bi_1−x_R_x_FeO_3_ (R = Ce, Tb; x = 0.05, 0.10 and 0.15) nanostructures.

The UV–Vis diffuse reflectance spectra (DRS) of Bi_1−x_R_x_FeO_3_ (R = Ce, Tb; x = 0.00, 0.05, 0.10 and 0.15) nanostructures are shown in Figure 4a,b and the band gaps deduced from Kubelka-Munk function [45]. The nanostructures exhibited a greater absorption in the visible range and the optical bandgap was found to be 2.03 eV [38] for BiFeO_3_ nanostructures. The observed band-gap energies [1.95, 1.91 and 1.82 eV for Bi_1−x_Ce_x_FeO_3_ (x = 0.05, 0.10 and 0.15)] are noticeably reduced with an increase of RE concentration. The obtained decreased bandgaps were found to be 2.00, 1.90 and 1.87 eV for the Bi_1−x_Tb_x_FeO_3_ (x = 0.05, 0.10 and 0.15) nanostructures, respectively.

The possible reduction in the bandgap could be ascribed to the RE-substitution induced formation of new energy states (defect levels) introduced between the valence band and conduction band [46]. Another perspective is the structural distortion persuaded modification in RE-modified BFO nanostructures. The bond angle variation in Fe-O-Fe due to the RE substitution also results in the reduced band-gap energy. The hybridization between Fe-3d and O-2p, in turn, depends on the Fe-O-Fe exchange angle and any change in the bond angle also alters the bandgap of BFO [46,47]. The narrow band gap values and high absorption in the visible range of BFO: Ce and BFO: Tb nanostructures leads the potential photocatalytic applications.

**Figure 5:**
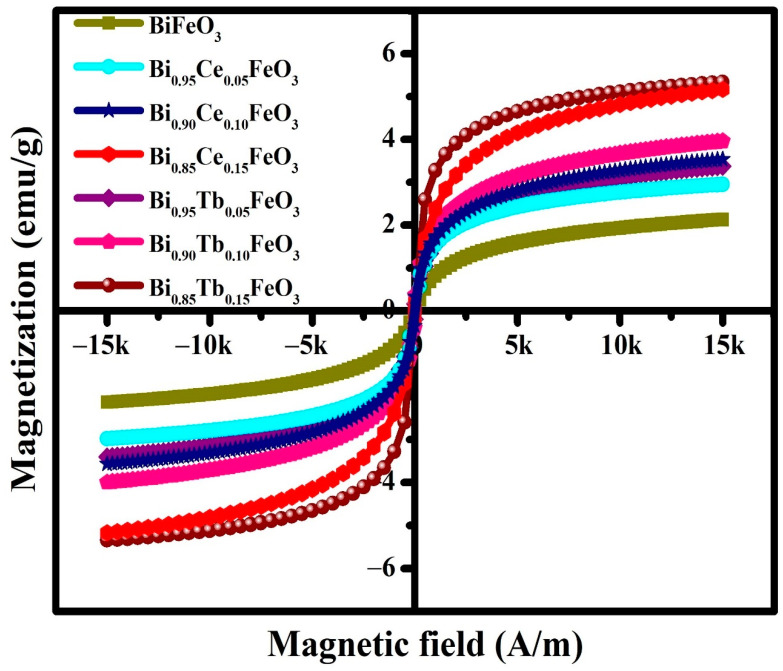
Magnetization (*M*) versus field (*H*) hysteresis loops for the prepared BFO: RE, measured at 300 K.

Figure 5 shows the magnetization-magnetic field (*M*-*H*) curves of BFO: RE nanostructures at room-temperature. A weak ferromagnetic behavior is observed for the x = 0.00 with spontaneous magnetization (*M_s_*) of 2.28 emu/g. By increasing the concentration of the RE in BFO, spontaneous magnetization was found to increase, exhibited *M_s_* values are 3.33, 3.93, 5.35, 2.94, 3.51 and 5.10 emu/g for Bi_0.95_Ce_0.05_FeO_3_, Bi_0.90_Ce_0.10_FeO_3_, Bi_0.85_Ce_0.15_FeO_3_, Bi_0.95_Tb_0.05_FeO_3_, Bi_0.90_Tb_0.10_FeO_3_ and Bi_0.85_Tb_0.15_FeO_3,_ respectively. The observed increment in magnetization for BFO:RE compositions are attributed to small particle size, a net magnetic moment of chosen RE element and the structural distortion [40,48].

**Figure 6:**
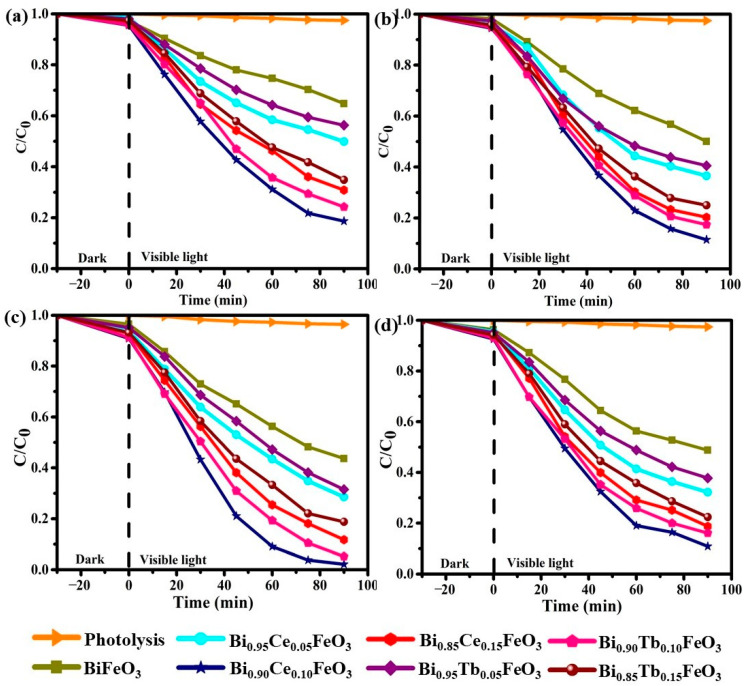
Photocatalytic degradation efficiencies of Bi_1−x_R_x_FeO_3_ (R = Ce, Tb; x = 0.00, 0.05, 0.10 and 0.15) at (**a**) 0.0T (**b**) 0.3T (**c**) 0.5 T and (**d**) 0.7T of external MF.

The photocatalytic degradation capabilities of PR over BFO: RE in the absence and presence of MFs (0.0, 0.3, 0.5 and 0.7T) under visible light illumination are shown in Figure 6a–d and summarized in Table 1. At the end of 90 min, the improved photodegradation efficiencies (35.2, 50.2, 81.3, 69.1, 43.7, 75.7 and 65.1% for BiFeO_3_, Bi_0.95_Ce_0.05_FeO_3_, Bi_0.90_Ce_0.10_FeO_3_, Bi_0.85_Ce_0.15_FeO_3_, Bi_0.95_Tb_0.05_FeO_3_, Bi_0.90_Tb_0.10_FeO_3_ and Bi_0.85_Tb_0.15_FeO_3_, respectively) are observed by increasing the RE content (Figure 6a). The degradation efficiencies increase with rising the RE concentration in BFO except for x = 0.15, and the highest efficiency was noticed for x = 0.10 in all the cases. This can be understood by doping the RE elements to BFO increases space-charge width near the phase boundary, that increases the number of separated electron and holes leads an enhanced photocatalytic activity [49]. The photocatalytic efficacies were increased gradually by applying the external MF. By applying the external MFs of 0.3T, the improved photodegradation ability have been observed for the compositions Bi_0.90_Ce_0.10_FeO_3_ and Bi_0.90_Tb_0.10_FeO_3_ (88.5% and 82.6%) compared with the absence of MF. A significant upward trend in the photocatalytic degradation ability of organic pollutant were noticed as 97.8% and 94.8% for Bi_1−x_R_x_FeO_3_ (R = Ce, Tb; x = 0.10) at 0.5T of MF. However, the decrement in degradation were observed at 0.7T as 89.1 and 85.9%. These results suggest the optimum MF to achieve higher photocatalytic efficiencies is 0.5T, which retards the recombination of charge carriers and improves carrier transport to the surface.

The enhancement in catalytic activity by applying the external magnetic fields can be ascribed to the manifestation of the magneto-hydrodynamic effect (MHD) [34], where the magnetic convection can be attributed to the effective Lorentz force [50] on charged species. This force may help to modify the scavenging rates of the excited electrons and separate the oppositely charged species efficiently, which generates more hydroxyl radicals to oxidize PR leads to higher degradation efficiencies in the presence of the external MF. Apart from this, the spin-state mixing can be affected by the MF explained through Δg mechanism [27]. According to the ΔgM, the MFs influence the spin conversion between S and T states of a radical pair [51,52]. The geminated singlet pairs of electrons and holes produced by irradiation, can recombine with one another. By applying the MFs, the singlet pairs of charge carriers undergo spin state mixing to produce triplet pairs, that cannot recombine. Thus, the increased yield of triplet pairs escape from the pairs and reacts with PR anion and cation radicals on the surface of the semiconductor, leads to enhancement in the catalytic activity at 0.3 and 0.5T of applied MFs. However, the observed decreased photocatalytic activity at MF of 0.7T, indicates that spin-state mixing can be blocked by higher MFs and accelerates the recombination of electrons and holes if the present MF-effects occur during the re-encounter of free radicals [53]. At high MFs, the free radical activity may decrease by shifting the triplet to singlets and participate in the recombination from the s-pairs, which is much faster than the escape process. i.e., triplet pairs can disappear by the recombination process instead of the escape one and decreases the efficiency of PR degradation.

**Figure 7:**
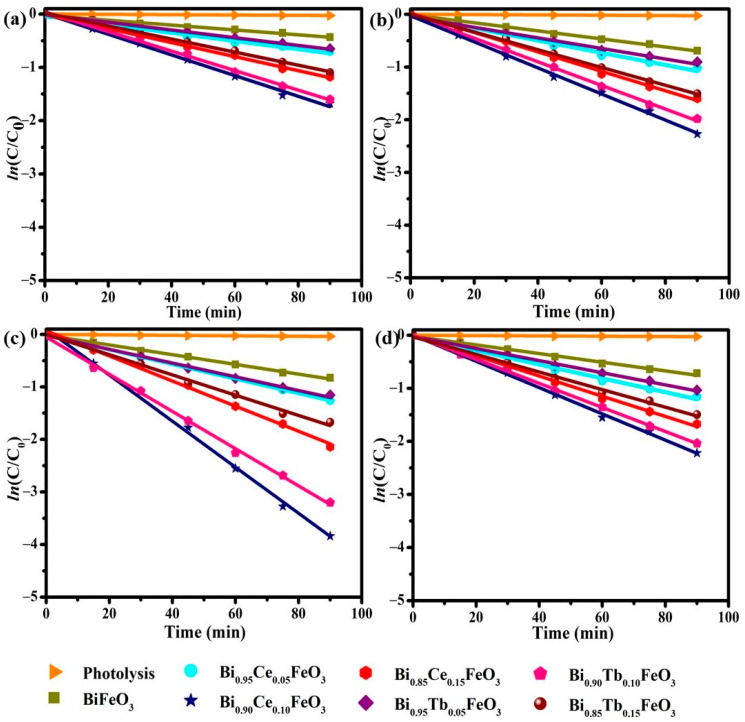
Photodegradation kinetics of Bi_1−x_R_x_FeO_3_ (R = Ce, Tb; x = 0.00, 0.05, 0.10 and 0.15) nanostructures at (**a**) 0.0T (**b**) 0.3T (**c**) 0.5T and (**d**) 0.7T of MF.

The PR degradation kinetics by BFO:RE nanostructures followed a pseudo-first-order reaction kinetics [54] shown in Figure 7a–d. The rate constants (*k*) were determined from the slope of the *ln*(*C/C*_0_) versus time (*t*), and tabulated in Table 2, signifies the enhanced catalytic activity was noticed at 0.5T for x = 0.10 in both the cases of RE substitution. 

**Figure 8:**
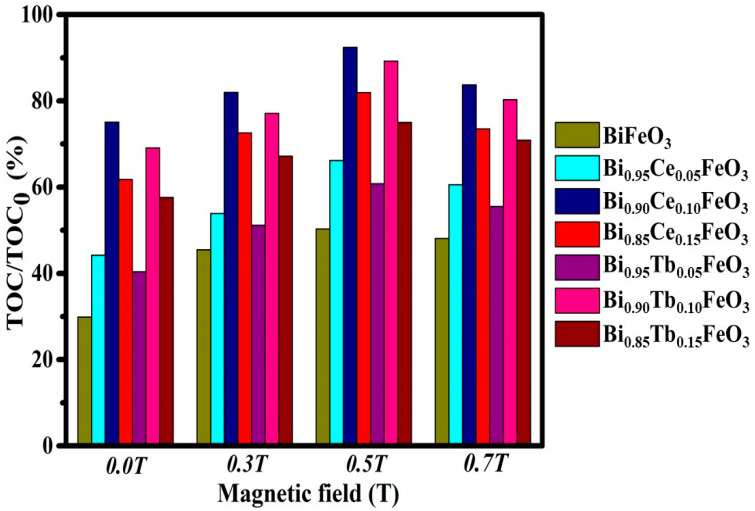
Mineralization efficiencies of BFO: RE nanostructures at different MFs.

Figure 8 shows the mineralization efficiencies of PR by BFO: RE nanostructures under different MFs. The obtained values for the mineralization degradation and their corresponding degradation efficiencies were tabulated in Table 1. For the composition x = 0.10, observed the highest mineralization efficiencies of 92.4% and 89.2% for BFO: Ce and BFO: Tb nanostructures at 0.5T of the MF, confirms no aromatic rings of organic carbon left and the effectiveness in degrading the PR during photocatalytic experiments.

Reproducibility is one of the tough issues in MF-assisted heterogeneous photocatalytic systems. To ensure the reproducibility, stability and reusability of BFO:RE, repetitive photodegradation studies were conducted for five consecutive cycles.

**Figure 9:**
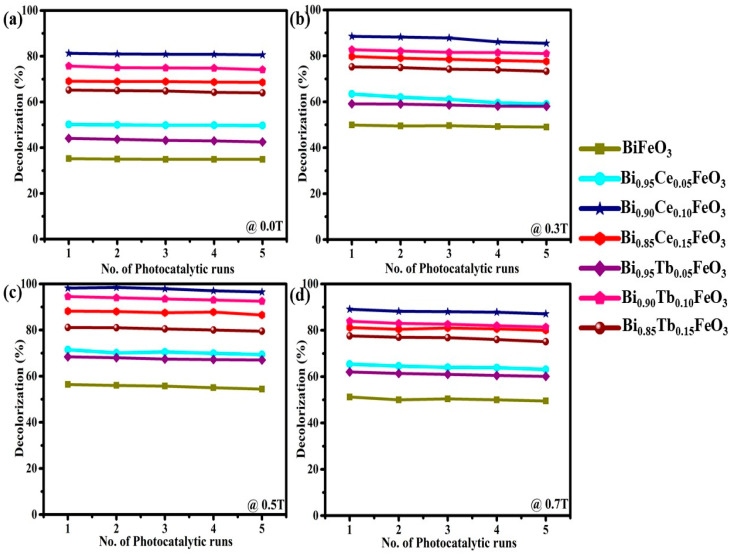
Recyclic degradation efficiencies of PR over BFO: RE nanostructures at (**a**) 0.0T (**b**) 0.3T (**c**) 0.5T (**d**) 0.7T of MF.

Figure 9a–d shows the efficiencies in the degradation of PR dye with an error less than 3% even after the five-sequential cycles, which validates the MF-assisted photocatalysis was certainly reproducible with highly reusable and stable BFO:Tb magnetic photocatalysts.

## 4. Conclusions

In summary, BFO: RE nanostructures were synthesized by the hydrothermal method. All the prepared samples were showed a nano-rod-like morphology, confirmed from the HR-TEM images. The structural studies showed the substitution induced structural transition from rhombohedral to orthorhombic with increasing the concentration of RE to BFO. The studies on the bandgap revealed that bandgap of the RE substituted BFO nanostructures decreased with increasing concentration of RE content. The enhanced magnetic properties were observed in BFO: RE nanostructures due to the substitution induced suppression of cycloidal spin structure. The investigations on photocatalytic studies revealed an enhanced photodegradation of PR was observed by applying the external magnetic field of 0.5T for the composition x = 0.10 in both the cases. The degradation raised to 97.8, 94.8% as magnetic strength increases from 0.0 to 0.5T and falls to 89.1%, 85.9% for 0.7T for Bi_1−x_R_x_FeO_3_ (R = Ce, Tb; x = 0.10), respectively. The enhanced degradation of PR in MF is attributed to magnetically stimulated hindering the recombination of photo-generated charge carriers. The recyclable tests were confirmed the prepared BFO: RE nanostructures are highly reusable, and the MF-assisted catalytic systems were reproducible.

## Figures and Tables

**Table 1:** Photodegradation and Mineralization efficiencies of PR over BFO: RE nanostructures at different magnetic fields.

Sample	Photodegradation Efficiencies (%)				Mineralization Efficiencies (%)			
	@0.0T	@0.3T	@0.5T	@0.7T	@0.0T	@0.3T	@0.5T	@0.7T
BiFeO_3_	35.2	49.9	56.4	51.2	29.9	45.5	50.3	48.1
Bi_0.95_Ce_0.05_FeO_3_	50.2	63.4	71.5	67.8	44.2	53.9	66.2	60.6
Bi_0.90_Ce_0.10_FeO_3_	81.3	88.5	97.8	89.1	75.1	82.0	92.4	83.7
Bi_0.85_Ce_0.15_FeO_3_	69.1	79.7	88.2	81.2	61.8	72.6	81.9	73.5
Bi_0.95_Tb_0.05_FeO_3_	43.7	59.4	68.5	62.2	40.4	51.2	60.8	55.5
Bi_0.90_Tb_0.10_FeO_3_	75.7	82.6	94.8	85.9	69.1	77.1	89.2	80.3
Bi_0.85_Tb_0.15_FeO_3_	65.1	75.0	81.2	77.6	57.6	67.2	75.0	70.9

**Table 2:** First-order reaction rate constants (*k*) at different magnetic fields for BFO: RE nanostructures.

Sample	@0.0T		@0.3T		@0.5T		@0.7T	
	*R* ^2^	*k* (min^−1^ × 10^−3^)	*R^2^*	*k* (min^−1^ × 10^−3^)	*R^2^*	*k* (min^−1^ × 10^−3^)	*R^2^*	*k* (min^−1^ × 10^−3^)
BiFeO_3_	0.98	4.5	0.99	7.6	0.99	9.2	0.99	8.1
Bi_0.95_Ce_0.05_FeO_3_	0.97	7.6	0.98	11.6	0.99	13.7	0.99	12.9
Bi_0.90_Ce_0.10_FeO_3_	0.99	19.4	0.99	24.7	0.99	43.8	0.99	28.7
Bi_0.85_Ce_0.15_FeO_3_	0.99	13.1	0.99	18.4	0.99	23.9	0.98	19.0
Bi_0.95_Tb_0.05_FeO_3_	0.99	7.2	0.98	10.0	0.99	13.0	0.99	11.4
Bi_0.90_Tb_0.10_FeO_3_	0.99	18.3	0.99	22.4	0.99	35.4	0.97	23.6
Bi_0.85_Tb_0.15_FeO_3_	0.99	12.2	0.99	16.8	0.99	19.3	0.98	17.1

## Data Availability

Data can be made available upon reasonable request.
